# Vascular Endothelial Growth Factor Receptor 2 (VEGFR-2) Plays a Key Role in Vasculogenic Mimicry Formation, Neovascularization and Tumor Initiation by Glioma Stem-like Cells

**DOI:** 10.1371/journal.pone.0057188

**Published:** 2013-03-11

**Authors:** Xiaohong Yao, Yifang Ping, Ying Liu, Kequiang Chen, Teizo Yoshimura, Mingyong Liu, Wanghua Gong, Chong Chen, Qin Niu, Deyu Guo, Xia Zhang, Ji Ming Wang, Xiuwu Bian

**Affiliations:** 1 Institute of Pathology and Southwest Cancer Center, Third Military Medical University, Chongqing, China; 2 Laboratory of Molecular Immunoregulation, Cancer and Inflammation Program, Center for Cancer Research, National Cancer Institute at Frederick, Frederick, Maryland, United States of America; 3 Department of Spinal Surgery, Daping Hospital, Third Military Medical University, Chongqing, China; 4 Basic Research Program, SAIC-Frederick, Frederick, Maryland, United States of America; University of Bari Medical School, Italy

## Abstract

Human glioblastomas (GBM) are thought to be initiated by glioma stem-like cells (GSLCs). GSLCs also participate in tumor neovascularization by transdifferentiating into vascular endothelial cells. Here, we report a critical role of GSLCs in the formation of vasculogenic mimicry (VM), which defines channels lined by tumor cells to supply nutrients to early growing tumors and tumor initiation. GSLCs preferentially expressed vascular endothelial growth factor receptor-2 (VEGFR-2) that upon activation by VEGF, mediated chemotaxis, tubule formation and increased expression of critical VM markers by GSLCs. Knockdown of VEGFR-2 in GSLCs by shRNA markedly reduced their capacity of self-renewal, forming tubules, initiating xenograft tumors, promoting vascularization and the establishment of VM. Our study demonstrates VEGFR-2 as an essential molecule to sustain the “stemness” of GSLCs, their capacity to initiate tumor vasculature, and direct initiation of tumor.

## Introduction

Glioblastoma (GBM) is one of the most vascularized tumors with increased microvasculature as a hallmark in pathology [1]. Previous studies of cancer vascularization focused mainly on the angiogenesis that is developed through sprouting from pre-existing vessels and the vasculogenesis that is established via recruitment of endothelial progenitor cells (EPCs) from the bone marrow [2]. Anti-vascular endothelial growth factor (VEGF) therapy has achieved limited efficacy in GBM due to rapidly developed resistance [3], [4]. Therefore, better understanding of the mechanisms of tumor vascularization may improve the efficacy of anti-antiangiogenic therapy.

Vascularization in brain tumors is a complex process that involves vessel co-option [5], angioblast vasculogenesis [6], intussusceptive microvascular growth [7] and vasculogenic mimicry (VM). VM defines the ability of highly invasive tumor cells to form fluid-conducing channels. The presence of VM in malignant tumors is associated with increased patient mortality [8], [9]. Morphologically, the channels of VM consist of a basement membrane with lining of tumor cells in the external wall, without endothelial cells (ECs) on the inner wall despite the presence of blood flow in the channels [10]. Microarray analysis of VM-positive tissues from aggressive melanoma reveals increased expression of genes associated with undifferentiated embryonic-like phenotype [11], suggesting the participation of cancer stem cells (CSCs), a subpopulation of tumor cells that possess the capacity of self-renewal, multi-lineage differentiation, tumor initiation and resistance to chemo- or radio-therapy [12]–[15].

CSCs have been identified in brain tumors, including GBM [15]–[18]. Glioma stem-like cells (GSLCs) can be enriched from established cell lines or primary tumor tissues by using CD133 positive selection or generating neurospheres in serum-free media containing growth factors [12], [19]–[21]. We and others have demonstrated that GSLCs actively interact with vascular niche in the tumor and promote angiogenesis through the release of VEGF to recruit and stimulate the proliferation of host ECs [19]–[21]. Moreover, recent studies have shown that GSLCs may directly participate in the formation of tumor vessel by transdifferentiating into vascular EC-like cells [22], [23]. The ability of GSLCs to acquire an EC-like phenotype and directly form tumor vasculature represents a novel mechanism of tumor vascularization [24], [25]. In addition, GSLCs may play a role in the formation of VM, which is important for growing tumors to obtain nutrients during the early stage of progression [26], [27]. In this study, we report that GSLCs expressed higher levels of VEGF receptor 2 (VEGFR-2), which was essential for controlling the self-renewal, tumorigenicity and the formation of new vessels and VM in tumors by GSLCs.

## Materials and Methods

### Cell Culture

The GBM cell line U87 (ATCC, VA, USA) was maintained as described [28], [29]. To obtain tumor spheres, U87 cells were plated in 25 cm^2^ flasks. After reaching 70% confluence, the cells were washed with PBS twice before further culture in stem cell medium consisting of DMEM containing bFGF (10 ng/ml) (PeproTech, NJ, USA), EGF (10 ng/ml) (PeproTech), B27-supplement (1∶50, Invitrogen, CA, USA), penicillin (100 U/ml )/streptomycin (100 U/ml) (Lonza, NJ, USA) in DMEM with nutrient mix F12 and glutamax (DMEM/F12) (Invitrogen). After 14 to 21 days when spheres grew to the size with a dark core under light microscopy, the spheres were dissociated by trypsin-EDTA (0.05%) for 5 minutes and single cell suspension was cultured in flasks at 1×10^5^ cells/ml. For cell differentiation, floating spheres were cultured in 6-well plates with DMEM/F12 containing 10% FCS (Lonza) for 7 days.

### Primary Human Glioma Samples

Glioma specimens were obtained from patients in the Department of Neurosurgery, Southwest Hospital, Third Military Medical University, Chongqing, and Tiantan Hospital, Beijing, China with written consent. The tumors were classified independently by at least two pathologists according to 2007 WHO classification of central nervous system tumors [30]. All experiments were approved by the institutional ethics committee.

### Antibodies

Antibodies used in the characterization of floating spheres included anti-human CD133 (1∶30 dilution. sc-30220, rabbit polyclonal, Santa Cruz Biotechnology, USA), anti-human nestin (1∶100 dilution. MAB5326, mouse monoclonal, Chemicon International, USA), anti-human Notch (1∶100 dilution. sc-23299, goat polyclonal, Santa Cruz Biotechnology, USA), anti-human Oct4 (1∶100 dilution. AB3209, rabbit polyclonal, Chemicon International, USA) and anti-human Nanog (1∶100 dilution. sc-30331, goat polyclonal, Santa Cruz Biotechnology, USA). Secondary antibodies used for detection were Cy3-conjugated goat anti-rabbit IgG (1∶50 dilution. AP187C, Chemicon International, USA) and FITC-conjugated goat anti-mouse IgG (1∶100 dilution. AQ303F, Chemicon International, USA).

The differentiated cells were characterized with the following antibodies: anti-human glial fibrillary acidic protein (GFAP) (1∶30 dilution. 610565, mouse polyclonal, BD Biosciences, USA), anti-human microtubule associated protein 2 (MAP2) (1∶200 dilution. AB5622, rabbit polyclonal, Chemicon International, USA), anti-human β-tubulin (1∶50 dilution. MAB1637, mouse monoclonal, Chemicon International, USA). Secondary antibodies used for detection were the same as described above.

Primary antibodies used for staining frozen sections were anti-human CD133 (1∶30 dilution. sc-30220, rabbit polyclonal, Santa Cruz Biotechnology, USA), anti-human VEGFR-2 (1∶100 dilution. #2479, rabbit monoclonal, Cell Signaling Technology, USA), anti-human VEGF (1∶100 dilution. 555036, mouse monoclonal, BD Biosciences, USA), anti-mouse CD31 (1∶50 dilution. 550274, rat monoclonal, BD Biosciences, USA), anti-human CD31 (1∶30 dilution. 550389, mouse monoclonal, BD Biosciences, USA). Primary antibodies were detected by goat anti-rabbit IgG Cy3 (1∶50 dilution. AP187C, Chemicon International, USA), rabbit anti-rat Alexa 549 (1∶500 dilution) and goat anti-mouse Alexa 549 (1∶500 dilution. Invitrogen, USA).

### Immunohistochemistry (IHC) and Histochemistry

Cells were fixed in 4% formaldehyde (Sigma-Aldrich, St. Louis, MO, USA) and frozen tumor sections were fixed with ice-cold acetone. The cells and frozen sections were blocked with 3% BSA (1 h, 37°C), and incubated with primary (overnight, 4°C) and secondary antibodies (30 minutes, 37°C). The cells were counterstained with 4-, 6- diamidino-2-phenylindole (DAPI at 10 µg/ml, Sigma-Aldrich) to mark nuclei.

Double immunofluorescence staining on frozen sections was used for detecting the co-expression of CD31 and GFAP or Laminin B2 (1∶100 dilution. 610722. mouse monoclonal, BD Biosciences, USA). The cells were incubated with anti-human CD31 overnight at 4°C. After revealing CD31 by Alexa 549, the cells were incubated with goat anti-human GFAP at 4°C overnight. GFAP in cells was detected by FITC-conjugated goat anti-mouse IgG. Isotype-matched IgGs were used as negative controls. Similar procedures were used for double immunofluorescence staining of laminin B2 and GFAP, VEGF and VEGFR-2 as well as CD133 and VEGFR-2.

CD34-PAS or nestin-PAS dual-staining on paraffin sections of human glioma specimens [31], and xenograft tumors formed byU87 cells and GSLCs was performed as follows: 4–5 µm paraffin sections were stained with anti-CD34 (1∶200 dilution. 550390, monoclonal mouse anti-human, BD Biosciences, USA) and anti-nestin antibodies. The sections were then stained with PAS and counterstained with Mayer’s hematoxylin followed by examination under light microscopy. ALP staining was visualized with a DAB Kit (DakoCytomation) and horseradish peroxidase staining was visualized with AEC (Maixin-Bio, China). Sections were counterstained with hematoxylin. To define matrix-associated vascular channels, GBM tissues were stained with PAS and hematoxylin. The sections were examined under light microscopy.

For kinase phosphorylation, floating spheres were kept in the differentiation medium without bFGF and EGF for 24 h before stimulation with 10 ng/ml VEGF for different times. Floating spheres were then laid on poly-lysine coated cover glasses and fixed in 4% formaldehyde. After washing in 0.01 M PBS and blocked with 5% BSA or normal goat serum for 1 h at 37°C in a humidified chamber, the cells were incubated with anti-human phospho-VEGFR-2 (Tyr1175)(1∶100 dilution. #2478, Cell Signaling Technology, USA ) followed by goat anti-rabbit IgG Cy3. Nuclei were counterstained with DAPI.

### Analysis of Microvessel Density (MVD)

MVD was assessed with anti-CD31 immunostaining (1∶100, BD Biosciences, USA), and a vessel was defined as any CD31 positive staining as previously described [32]. Briefly, after immunostaining, microscopic scanning of the entire tumor section under low power fields (× 40) of a laser scanning microscopy (Zeiss laser scanning microscopy 510 META, Carl Zeiss AG, Germany) was performed to identify hot spots that are the areas of highest neovascularization. Individual image was then taken under high power fields (× 200) to count CD31 positive cells or cell clusters in a defined area. In order to determine the mean number of MVD within a tumor, the number of CD31 positive cells or cell clusters was determined in five different areas with adjacent fields by two independent pathologists who had no knowledge of the sample identity. Final results of MVD was calculated by Image J 1.43u (NIH, USA).

### RT-PCR and Real-time Quantitative (q) RT-PCR

Total RNA was extracted from U87 GBM cells and GSLCs with an RNeasy mini kit (Qiagen, Valencia, CA). Primer sequences and PCR conditions are listed in Table S1. RT-PCR products were electrophoresed in 1% agarose and visualized by ethidium bromide staining. qRT-PCR was performed on an ABI PRISM 7900HT sequence detection system (Applied Biosystems, Norwalk, CA) using SYBR Green PCR Master Mix (Applied Biosystems). Primer sequences and qRT- PCR conditions are listed in Table S2. Standard curves were generated and the relative amount of target mRNA was normalized against β-actin mRNA.

### Western Blotting

GSLCs cultured in differentiation medium for 24 h were stimulated with VEGF. The cells were lysed on ice in 100 µl RIPA buffer with a protease and phosphatase inhibitor cocktail (Pierce Biotechnology, PA, USA). After centrifugation at 10,000×g at 4°C for 20 min, equal amount of proteins underwent 4% to 12% Bis-Tris SDS-PAGE (Invitrogen). The proteins were then blotted onto Immobilon-P PVDF membranes (Millipore, CT, USA). After blocking with 3% nonfat dry milk for 1 h at room temperature, the membranes were incubated with primary antibodies in PBS containing 0.01% Tween 20 overnight at 4°C. The phosphorylated proteins were visualized by staining the membranes with horseradish peroxidase-conjugated secondary antibody for 1 h followed by incubation with Super Signal Chemo-luminescent Substrate Stable Peroxide Solution (Pierce) and detection with BIOMAX-MR film (Eastman Kodak). When necessary, the membranes were stripped with Restore Western Blot Stripping Buffer (Pierce) and reprobed with antibodies against other proteins.

### Chemotaxis

Cell chemotaxis was measured in 48-well chemotaxis chambers (NeuroProbe, Cabin John, MD). A 27 µl aliquot of chemoattractants was placed in the wells of the lower compartment and 50 µl of tumor cells (at 2×10^6^/ml) were placed in the wells of the upper compartment. Two compartments of the chamber were separated by a 10 µm pore-sized polycarbonate filter (GE Osmonics Labstore) coated with 50 µg/ml collagen type I (BD). After incubation at 37°C for 240 min, the filters were removed, stained, and cells that migrated across the filters were counted under light microscopy. The results were expressed either as the mean number (± SE) of migrated cells in three high-powered fields (400×) in triplicates or as chemotaxis index that represents the fold increase in cell migration in response to stimulants over medium control.

### In vitro Tubule Formation

Vasculogenic tubule formation by GBM cells was tested on the reduced growth factor matrix (Matrigel, BD). Eight-chamber slides (Lab-Tek, 155411) were pre-coated with Matrigel (0.1 ml/well, BD) and incubated at 37°C for 30 min. Floating spheres of GSLCs were resuspended in the EC basal medium-2 (EBM-2, Lonza) containing 2% FCS with or without 10 ng/ml VEGF (PeproTech) and 1 µg/ml VEGFR-2 blocking antibody (sc-74002, Santa Cruz Biotechnology, USA). After 4 days, the tubules formed by GSLCs were counted under light microscopy. Tubule formation was analyzed by Image J software [33].

### Cell Proliferation

Tumor cells were cultured in 96-well plates at 2×10^4^ per well in DMEM containing 10% FCS for 12 h. The cells were then incubated in 100 µl DMEM containing 0.5% FCS at 37°C for different times. Cell growth was measured by colorimetric assay using 3-(4, 5-dimethylthiazol-2-yl)-5(3-carboxymethonyphenol)-2-(4-sulfophenyl)-2H-tetrazolium (MTS) (Promega, Madison, WI). The reduction in MTS, which reflects the number of viable cells per well, was measured after 3 h at the absorbance of 490 nm.

### Transfection of U87 GBM Cells with VEGFR-2 shRNA

Oligonucleotides encoding shRNA targeting VEGFR-2 were designed and cloned into a pGFP-V-RS plasmid vector (TG320400, OriGene, MD, USA). Lipofectamine RNAiMAX Reagent (Invitrogen) was used for transfection of plasmid into U87 GBM cells. Cells stably expressing VEGFR-2 shRNA were selected and maintained in medium containing 2 µg/ml puromycin (Sigma-Aldrich).

### Tumor Xenograft Implantation

U87 GBM cells at 2×10^6^ and GSLCs at 1×10^4^ in 100 µl PBS were subcutaneously implanted into the flank of 4-week-old (20–22 g) female athymic Ncr-nu/nu mice (NCI-Frederick Cancer Research Facility). Tumor volume (T_V_) was calculated by the formula: T_V_ = l (length)×w^2^ (width)/2 [19]. When the width or length of xenograft tumors reached 2 cm, mice were sacrificed and frozen tumor sections were made. Animal care was provided in accordance with the Guide for the Care and Use of Laboratory Animals.

CD133 positive cells are in red fluorescence. Microscopic scanning of the entire tumor section was scanned in low power fields (x 40) by a laser scanning microscopy (Zeiss LSM 510 META) to identify the areas of the cells with highest red fluorescence. Individual image was then taken in high power fields (x 200) to count CD133 positive cells. In order to determine the mean value, the number of CD133 positive cells was determined in five different areas in adjacent fields. Image J 1.43u (NIH, USA) was applied to calculate the number of CD133 positive cells.

### Transmission Electron Microscopy (TEM)

U87 and GSLC xenograft tumors were fixed in 2.5% buffered glutaraldehyde and were post-fixed in a solution of 1% osmium tetroxide, dehydrated, and embedded. Thin sections were stained with uranyl acetate-lead citrate and examined with a Hitachi H-7500 transmission electron microscope.

### Statistical Analyses

All experiments were conducted at least three times with reproducible results. Results presented were from representative experiments. Where applicable, data were expressed as the mean ± SE. Statistical significance of the difference between testing and control groups was analyzed with SPSS10.0 software. When two groups were compared, the unpaired Student’s *t* test was used. When multiple groups were evaluated, one-way ANOVA was used. *P*<0.05 was considered statistically significant.

## Results

### GSLC-derived EC-like Cells form VM and Neovasculature in Xenograft Tumors and Primary Human GBM

Our previous studies have demonstrated the existence of GSLCs in the human GBM cell line U87 [19]–[21]. GSLCs were enriched in serum-free culture medium as evidenced by sphere formation (**Fig. S1A**). GSLCs obtained were positive for CD133 and a neural precursor marker nestin as well as multiple stem cell markers including Oct4, Nanog and Notch1. Additionally, GSLCs cultured in differentiation medium expressed GFAP for glial cells, neuronal class III beta-tubulin and microtubule associated protein 2 (MAP2) for neurons (**Fig. S1B, C**). Moreover, GSLCs exhibited markedly increased capacity to initiate tumors when implanted *s.c.* in nude mice. Also, co-expression of CD133 and VEGFR-2 was detected in the tumors formed by U87 GBM cells and GSLCs (**Fig. S2A–C**). However, GSLC-derived tumors contained increased number of CD133^+^/VEGFR-2^+^ cells as compared to the tumors formed by parental U87 GBM cells (**Fig. S2C**, *right*). Therefore, U87 GBM cells contain GSLCs with typical stem cell properties and the expression of an EC marker VEGFR-2.

**Figure 1:**
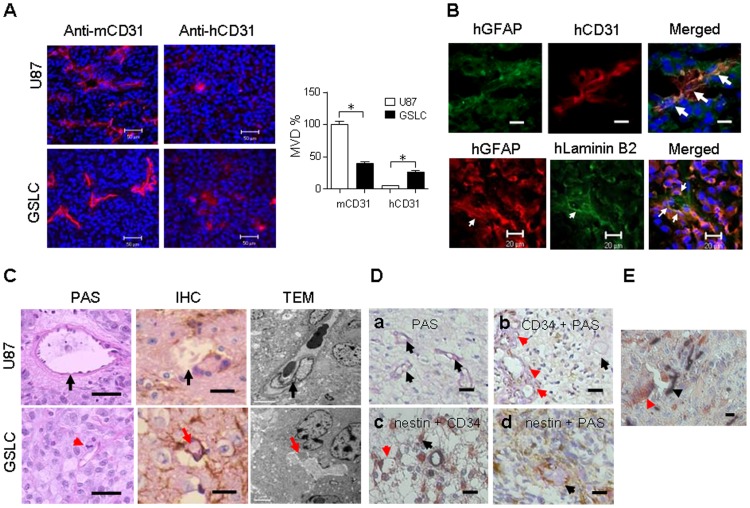
Vasculogenesis and VM in xenograft tumors derived from GSLCs and primary human glioma. (**A**) ECs detected with anti-human and anti-mouse CD31 antibodies in the tumors formed by U87 GBM cells (*top*) and GSLCs (*bottom*). Right panel shows quantification of human (h) and mouse (m) CD31^+^ cells. * Indicates statistically significant differences between tumors formed by U87 parent cells and GSLCs (* *p<0.01*). Nuclei were counterstained by DAPI (blue). Scale bar = 50 µm. (**B**) Double-immunofluorescence staining to detect human CD31^+^ (red) and GFAP^+^ (green) (*top*) as well as human GFAP (red) and laminin B2 (green) (*bottom*) in GSLC-derived xenograft tumors. Nuclei were counterstained by DAPI (blue). Scale bar = 20 µm. (**C**) PAS: Vascular basement membrane stained by PAS in xenograft tumor sections. Black arrow indicates blood vessel lined by ECs. Red arrow indicates VM lined by glioma cells with mitosis. Scale bar = 50 µm. IHC: Double positive staining of nestin and PAS formed tubule including red cells in GSLC xenograft tumor section, but not in U87 xenograft tumor section. Black arrow indicates blood vessel lined by ECs which were PAS-positive reaction only in the basement membrane. Red arrow indicates VM lined by glioma cells positive in nestin. Scale bar = 50 µm. TEM: Transmission electron microscopy of VM in GSLC initiated xenograft tumors. A vascular channel is lined by a thin basal lamina (red arrow) corresponding to the walls of the channel seen by conventional light microscopy. No endothelial cells line the tubule as compared to xenograft tumor formed by U87 cells (upper panel). Scale bar = 5 µm. (**D**) ECs in a human GBM section detected by anti-CD34 (black color). Vascular basement membrane with PAS staining (purple magenta) and tumor cells are labeled with anti-nestin antibody (brown color). **a**, Black arrows show tubular blood vessels stained with PAS. **b**, Double-staining of CD34 and PAS shows vessels (red arrows); PAS-positive tubules are lined by CD34^−^ cells (black arrow). **c**, Tumor vessels containing red blood cells are positive for nestin but negative for CD34 (red arrow). Black arrow shows CD34^+^ blood vessels. **d**, PAS positive tubules containing red blood cells are lined by nestin-positive cells on the luminal surface (black arrow). Scale bar = 50 µm. (**E**) Tumor cell-lined vessels (red arrow) and EC-lined vessels (black arrow) are detected in the same tubule in a human GBM specimen. Scale bar = 20 µm.

We and others have shown that CSCs produce higher levels of VEGF [6], [16]–[19] to induce the formation of capillary-like structures by ECs [16]. To investigate the capability of GSLCs to initiate new blood vessels in tumor, we compared the sources of ECs in both GSLC- and the parental U87 GBM cell-derived xenograft tumors. **Figure 1** shows that both U87- and GSLC-derived tumors contained murine EC-like cells as stained by an anti-mouse CD31 antibody (**Fig. 1A**, *left*). Both U87- and GSLC-derived tumors also contained human EC-like cells as stained by an anti-human CD31 antibody (**Fig. 1A**, *middle*). However, GSCL-derived tumors contained a higher number of human EC-like cells than parental U87-derived tumors. In GSLC-derived tumors, 34% of CD31^+^ cells were of the human origin, whereas almost all CD31^+^ cells in the tumors formed by parental U87 GBM cells were from mice (**Fig. 1A**, *right*).

The presence of human EC-like cells in GSLC-derived tumors was confirmed by co-staining of tumor tissues in which a proportion of CD31^+^ cells also expressed the glial marker GFAP (**Fig. 1B**, *top*), consistent with the findings in primary human GBM [34]. We further observed the co-expression of human laminin B2 and GFAP in GSLC-derived tumors (**Fig. 1B**, *bottom*). These observations suggest the presence of GSLC-derived VM in GSLC-xenograft tumors. To confirm this, we detected red blood cells containing PAS-positive tubular structures lined by GBM cells with mitosis in GSLC-derived tumors. Moreover, to definitely demonstrated the formation of VM formed by tumor cells, by transmission electron microscopy we show in Fig1 C that VM in GSLC formed xenograft tumors was lined by mural cells of tumor, but not endothelial origin. In contrast, parental U87 cell-derived tumors contained vessels with PAS-positive reaction only in the basement membrane, which were coated with endothelial cells as defected in both IHC and TEM (**Fig. 1C**).

To identify VM in primary human GBM sections, we used PAS and antibodies against CD34 and nestin to stain the tumor blood vessels. PAS-positive lumina were detected in human GBM sections (**Fig. 1D** **a**, black arrows). Tumor vessels were positive for both CD34 and PAS (**Fig. 1D** **b**, red arrows). In the same tumor sections, we detected PAS-positive tubular structures lined by CD34^−^ cells in the luminal surface (**Fig. 1D** **b**, black arrow). To examine whether these CD34^−^ cells were tumor cells, we double-stained CD34 and nestin. **Figure 1D** **c** shows that CD34^−^ cell-lined tubular structures were nestin-positive and contained red blood cells (**Fig. 1D** **c**, red arrow). In addition, PAS-positive tubular structures containing red blood cells were lined by nestin positive cells in the luminal surface (**Fig. 1D** **d**, black arrow). The interface of tumor cell-lined vessels and EC-lined vessels was also found in human GBM sections (**Fig. 1E**), with some tubular structures formed by both tumor cells (red arrow, brown color) and ECs (black arrow, black color). Thus, non-EC-lined vessels in GSLC-derived murine xenograft tumors are VM formed by tumor cells derived from human GSLCs and such VM can also be found in human primary GBM.

### GSLCs Preferentially Express VEGFR-2

**Figure 2:**
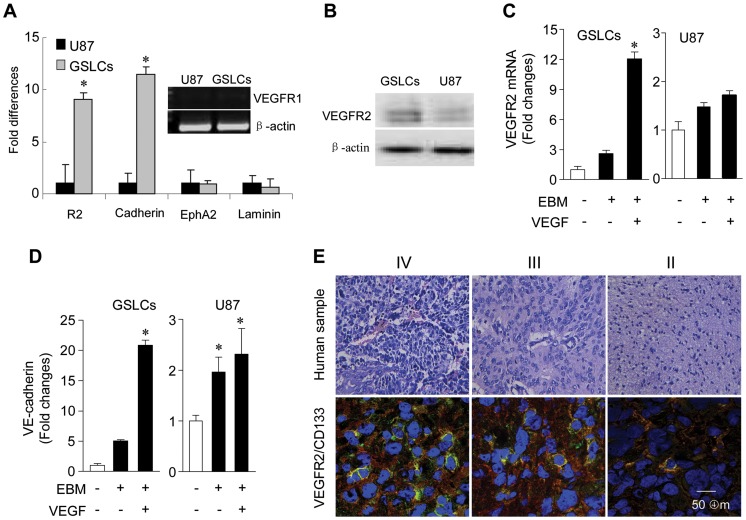
Preferential expression of VEGFR-2 by GSLCs isolated from U87 GBM cells. (**A**) The expression of mRNAs for VEGFR1, VEGFR-2, VE-cadherin, EphA2, and laminin 5γ2 in GSLCs and U87 GBM cells was measured by RT-PCR or real-time RT-PCR. * Indicates significantly increased expression by GSLCs. (**B**) Western blot of VEGFR-2 (230 and 200 KDa) in GSLCs and U87 GBM cells. β-actin was used as an internal control. (**C**, **D**) The effect of VEGF on the expression of mRNAs for VEGFR-2 (**C**) and VE-cadherin (**D**) in GSLCs or U87 GBM cells was measured by real-time RT-PCR. * Indicates significantly increased expression of genes compared to U87 cells or by VEGF treated cells (* *P<0.01*). (**E**) HE staining of human glioma specimens. IV: WHO Grade IV; III: WHO Grade III; II: WHO Grade II. Co-expression of CD133 (red) and VEGFR-2 (green) by human Grade IV GBM, Grade III anaplastic astrocytoma and Grade II astrocytoma sections is shown. Scale bar = 50 µm.

We next attempted to identify genes that may contribute to the VM-forming and vasculogenic property of GSLCs. Several genes involved in vasculogenesis and VM formation were expressed in GSLCs, including VEGFR-2, VE-cadherin, EphA2 and laminin 5γ2 (**Fig. 2A**). In particular, VEGFR-2 was more highly expressed in GSLCs at both mRNA and protein levels as compared to the parental U87 GBM cells (**Fig. 2A, B**). Notably, neither GSLCs nor U87 GBM cells expressed VEGFR1 (**Fig. 2A**, *insert*). Stimulation of GSLCs with VEGF upregulated the expression of the genes for VEGFR-2 (**Fig. 2C**) and VE-cadherin (**Fig. 2D**), consistent with the expression of both CD133 and VEGFR-2 in cells of primary human grade IV (12/15), III (6/10), II (1/7) glioma specimens (**Fig. 2E**). Therefore, VEGFR-2 is expressed by GSLCs derived from both GBM cell lines and primary GBM tissues.

### VEGFR-2 Expressed by GSLCs is Functional

**Figure 3:**
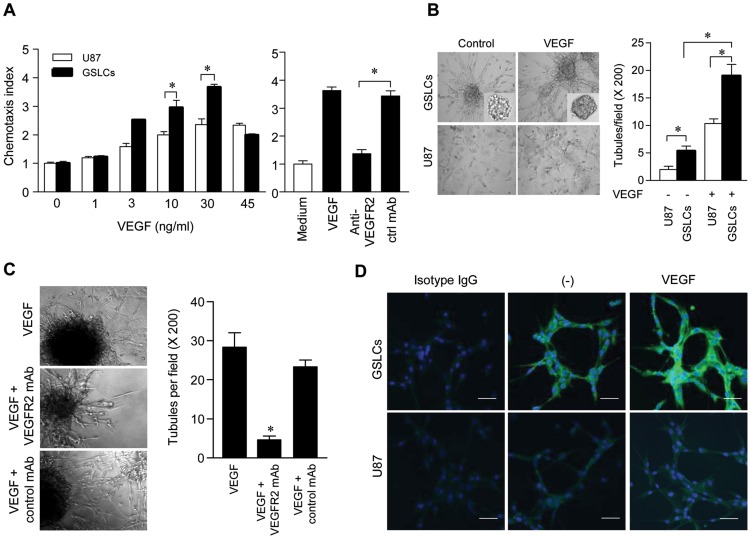
The function of VEGFR-2 in GSLCs. (**A**) VEGF-induced chemotactic of GSLCs (*left*). * Indicates significantly increased response shown by GSLCs compared to U87 parental cells (*p<0.05*). A VEGFR-2 neutralizing mAb blocked GSLC response to VEGF (*right*). Control mAB: ctrl mAb. * Indicates significantly reduced cell response in the presence of anti-VEGFR-2 (*p<0.05*). (**B**) Tubule formation by GSLCs treated by VEGF. **B**, *Left*: Vessel formation by GSLCs; *Right*: Quantitative analysis of tubule formation by GSLCs. Data represent the mean ± SEM in triplicates. * Indicates significantly increased tubule formation by GSLCs compared to U87 cells and cells treated with VEGF versus untreated cells (*p<0.05*). (**C**) The effect of anti-VEGFR-2 mAb on tubule formation by GSLCs (*left*). The black ball indicates sphere. *Right*: Quantitation of tubules. * Indicates significantly reduced tubule formation by GSLCs treated with anti-VEGFR-2 mAb compared to control cells (*p<0.05*). (**D**) VEGFR-2 expression on the tubules formed by VEGF-treated GSLCs.

VEGFR-2 expressed by GSLCs is functional, because VEGF induced significant chemotaxis of GSLCs and the response was more potent than the parental U87 GBM cells that expressed lower level of VEGFR-2 (**Fig. 3A**, *left*). Also, a VEGFR-2 mAb significantly reduced GSLC chemotaxis response induced by VEGF (**Fig. 3A**, *right*). In addition, GSLCs formed a higher number of tubules than the parental U87 GBM cells in the absence or presence of VEGF (**Fig. 3B**). The formation of tubule-like structures by GSLCs in response to VEGF was inhibited by the VEGFR-2 mAb (**Fig. 3B**, *right* and **C**). Further, VEGF stimulation increased the expression of VEGFR-2 by GSLCs (**Fig. 3D**). These results indicate an important role of VEGFR-2 in mediating the VM-forming and vasculogenic potential of GSLCs.

**Figure 4:**
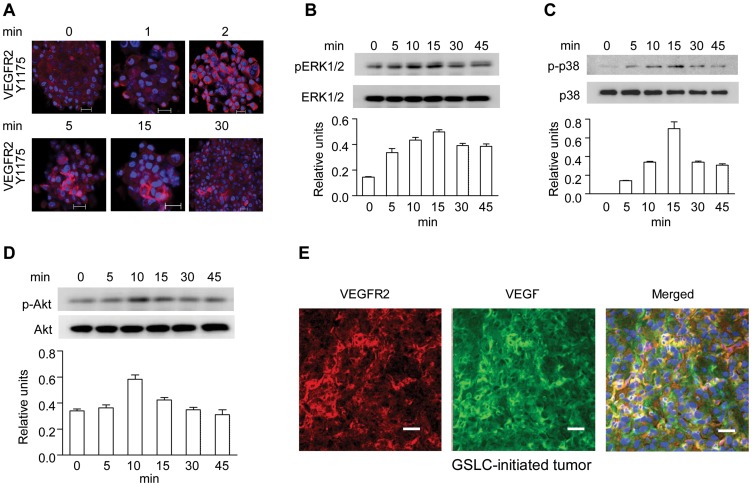
VEGF-induced phosphorylation of VEGFR-2, ERK, p38 and PI3K in GSLCs. (**A**) IF images of phosphorylation on Y1175 in VEGFR-2 in GSLCs stimulated by VEGF (10 ng/ml) for different minutes (min). Scale bar = 20 µm. (**B–D**) Western blot of ERK, p38 and AKT phosphorylation in GSLCs treated with VEGF (10 ng/ml) for different times (min). (**E**) IF double-staining of VEGFR-2 and VEGF in GSLC-initiated xenograft tumors. Scale bar = 20 µm. VEGFR-2 (red), VEGF (green).

Since VEGFR-2 phosphorylation on key tyrosine residues is necessary for activation of down-stream signaling molecules, we examined the phosphorylation of Y1059 and Y1175 in the C-terminal domain of VEGFR-2 [35]. There was a constitutive level of Y1059 phosphorylation in VEGFR-2 in GSLCs, which was not affected by VEGF stimulation [data not shown], suggesting self-phosphorylation at Y1059. In contrast to Y1059, Y1175 in VEGFR-2 was rapidly and transiently phosphorylated when GSLCs were stimulated with VEGF (**Fig. 4A**). The phosphorylation of Y1175 in VEGFR-2 was associated with increased activation of ERK1/2 and p38 MAPKs as well as AKT (**Fig. 4B, C, D**), which are known to be coupled to VEGFR-2 signaling pathway. The possibility that VEGFR-2 on GSLCs may be activated by ligand present in the tumor environment was confirmed in the xenograft tumors initiated by GSLCs in which tumor cells expressed high levels of both VEGFR-2 and VEGF (**Fig. 4E**). These results indicate that functional VEGFR-2 expressed by GSLCs mediates important GSLC functions such as increased cell migration and transdifferentiation into cells to form VM in tumor.

### VEGFR-2 is Critical for the Self-renewal and Tubule Formation by GSLCs

**Figure 5:**
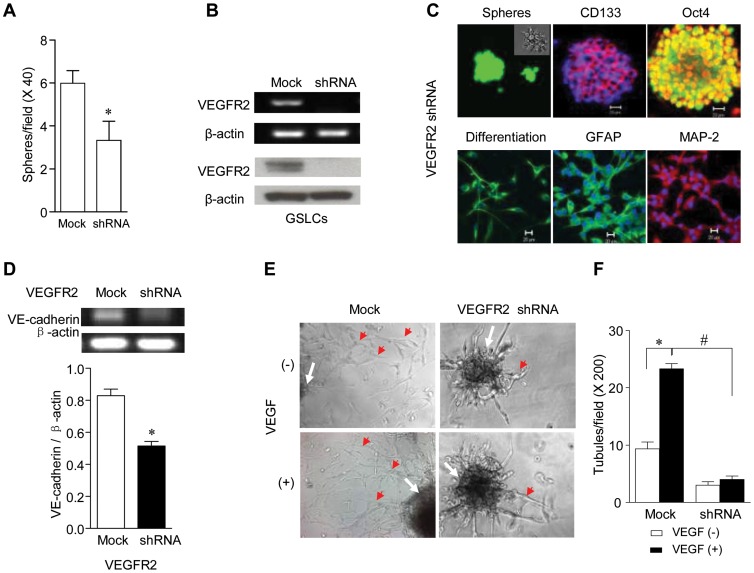
The effect of VEGFR-2 shRNA on the self-renewal of GSLCs and their formation of tubules. (**A**) Formation of spheres by GSLCs with VEGFR-2 shRNA. * Indicates significantly reduced sphere formation by U87 cells containing VEGFR-2 shRNA (*p<0.05*). (**B**) VEGFR-2 knockdown by shRNA in GSLCs. RT-PCR analysis of VEGFR-2 mRNA (*top*) and Western blot of VEGFR-2 (*bottom*). (**C**) IF images of spheres formed by VEGFR-2 knockdown GSLCs (*upper*) or by differentiated VEGFR-2 knockdown GSLCs (*lower*). IF staining of CD133 (red) and Oct4 (yellow) on spheres is shown in the upper panels. IF staining of GFAP (green) and MAP-2 (red) on differentiated cells is shown in lower panels. (**D**) RT-PCR of mRNA for VE-cadherin in GSLCs with VEGFR-2 shRNA. * Indicates significantly reduced mRNA in VEGFR-2 knockdown GSLCs compared to Mock shRNA cells (*p<0.05*). (**E**) Tubule formation on Matrigel by GSLCs with VEGFR-2 shRNA in the presence or absence of VEGF. Spheres are indicated by white arrows; tubules are indicated by red arrows. Images were taken under light microscopy (×200). (**F**) Quantitative analysis of tubule formation by VEGFR-2 knockdown GSLCs. * Indicates significantly increased tubule formation by GSLCs containing Mock shRNA in response to VEGF (10 ng/ml) (* *p<0.05*). # Indicates significantly reduced tubule formation by GSLCs containing VEGFR-2 shRNA.

To further clarify the function of VEGFR-2 in GSLCs, we used shRNA to knockdown VEGFR-2. **Figure 5A** shows that transduction of VEGFR-2 shRNA into the GBM cell line U87 reduced VEGFR-2 mRNA expression. Knockdown of VEGFR-2 markedly reduced the chemotaxis of U87 GBM cells in response to VEGF (**Fig. S3B**) and also reduced the proliferation of U87 GBM cells *in vitro* as compared with the wide type (WT) and mock knockdown cells (**Fig. S3C**). When subcutaneously injected into nude mice, VEGFR-2 knockdown GBM cells showed a markedly reduced tumorigenicity (**Fig. S3D**) with increased mouse survival (**Fig. S3E**).

Since we have shown that VEGFR-2 is more highly expressed in GSLCs enriched from parental U87 cells, we hypothesized that the marked suppression of the growth and tumorigenicity of U87 GBM cells by VEGFR-2 knockdown may be caused by a selective effect on GSLCs. We therefore examined the capacity of VEGFR-2 knockdown U87 GBM cells to form floating spheres, an indication of GSLC enrichment and self-renewal. The number of floating spheres derived from VEGFR-2 knockdown U87 GBM cells was significantly reduced, indicating that VEGFR-2 knockdown impaired the enrichment and self-renewal of GSLCs (**Fig. 5A**). **Figure 5B** shows that VEGFR-2 expression was absent in the GSLCs enriched from VEGFR-2 knockdown parental U87 cells. However, these GSLCs retained the expression of stem cell markers CD133 and Oct4 (**Fig. 5C**, *top*). When cultured in differentiation media, the cells were capable of converting into GFAP^+^ astrocytic and MAP-2^+^ neuronal cells (**Fig. 5C**, *bottom*).

Despite the expression of stem cell markers and the capacity of multi-lineage differentiation, VEGFR-2 knockdown reduced the expression of VM-associated marker VE-cadherin by GSLCs (**Fig. 5D**) and markedly inhibited their formation of tubules (**Fig. 5E**, *top* and **Fig. 5F**) in the presence or absence of VEGF stimulation (**Fig. 5E, F**). This is in contrast to VEGFR-2 expressing GSLCs, which maintained the capacity to form tubules and were highly responsive to VEGF stimulation (**Fig. 5E, F**). These results indicate that VEGFR-2 is essential for the self-renewal and formation of tubular structures by GSLCs.

### VEGFR-2 is Required for New Vascularization and Tumor Initiation by GSLCs

**Figure 6:**
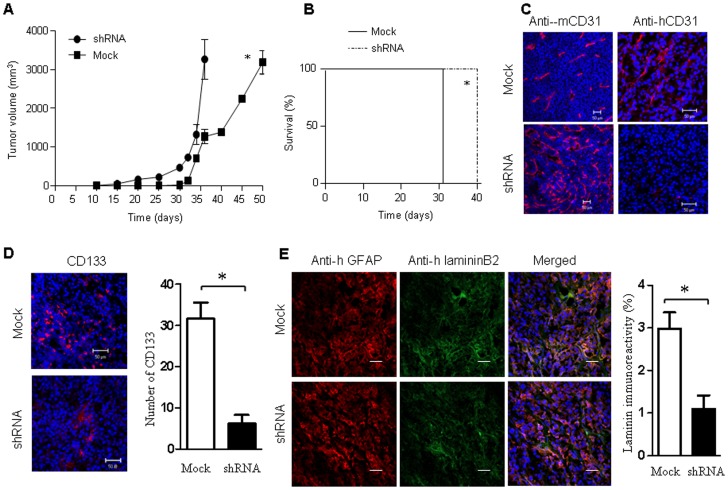
The effect of VEGFR-2 shRNA on tumorigenesis, angiogenesis, self-renewal and VM formation by GSLCs. (**A**) The growth of xenograft tumors initiated by GSLCs with or without VEGFR-2 shRNA. * Indicates significantly reduced growth of tumors formed by GSLCs with VEGFR-2 shRNA (*p<0.05*). (**B**) Survival of mice with xenograft tumors formed by GSLCs with or without VEGFR-2 shRNA. * Indicates significantly prolonged survival of mice bearing tumors formed by VEGFR-2 containing GSLCs (*p<0.05*). (**C**) IF images of murine or human CD31 (red) in the xenograft tumors formed by GSLCs with or without VEGFR-2 shRNA. Nuclei were stained with DAPI (blue). Scale bar = 50 µm. (**D**) Self-renewal of GSLCs with VEGFR-2 shRNA. IF staining of CD133 (red) in the xenograft tumors derived from GSLCs with or without VEGFR-2 shRNA. Nuclei were stained with DAPI (blue). Scale bar = 50 µm. * Indicates significantly decreased number of CD133-positive cells in mice bearing tumors formed by VEGFR-2 knock-down GSLCs (*p<0.05*). (**E**) VM formation by GSLCs with or without VEGFR-2 shRNA. IF staining of human LamininB2 (red) or human GFAP (green) in the xenograft tumors derived from GSLCs with or without VEGFR-2 shRNA. Nuclei were counterstained with DAPI (blue). Scale bar = 20 µm. Quantitative image analysis of laminin VM immunoreactivity for glioma derived from Mock or VEGFR-2 shRNA-transfected GSLCs xenografts (n = 6 recipient mice per experimental group). Y-axis, percentage of area with reactivity (mean ±SE, * *P*<0.01).

The reduced self-renewal and tubule formation by VEGFR-2 knockdown GSLCs and the reduced tumorigenicity of VEGFR-2-knockdown U87 parental cells led us to further test the tumorigenicity of GSLCs and their contribution to tumor vascularization in vivo. As compared to VEGFR-2-positive GSLCs, VEGFR-2-knockdown GSLC-formed tumors grew much more slowly in nude mice with prolonged mouse survival (**Fig. 6A, B**). Additionally, VEGFR-2 knockdown GSLC-derived xenograft tumors contained fewer vessels formed by cells of human origin as demonstrated by observations in which human CD31-positive vessels were hardly visible in the tumors formed by VEGFR-2 knockdown GSLCs. Rather, these tumors contained a large number of murine CD31-positive vessels (**Fig. 6C**). In contrast, VEGFR-2-positive GSLC-formed tumors grew much more rapidly and contained vessels stained for both human and murine CD31 (**Fig. 6A–C**). In addition, CD133-positive cells were markedly reduced in the tumors formed by VEGFR-2-knockdown GSLCs as compared to the tumors derived from VEGFR-2 positive GSLCs (**Fig. 6D**). These results indicate that VEGFR-2 actively participates in vasculogenesis and self-renewal of GSLCs in xenograft tumors.

To evaluate the role of VEGFR-2 in VM formation by GSLCs, we stained human GFAP and lamininB2 in tumors formed by GSLCs with or without VEGFR-2 shRNA. VM within xenografts were measured by quantitative image analysis [36] to assess the density of lamininB2 immunoreactivity per cross-section area. In tumors formed by GSLCs with VEGFR2 shRNA, VM was inhibited by 60% **(** **Fig. 6E** **)** compared with tumors originated from mock transfected GSLCs [1.2%±0.5% versus 3.0%±0.9% (n = 5)]. These results indicate that VEGFR-2 is critical for VM formation by GSLCs and the rapid growth of tumors initiated by GSLCs.

## Discussion

In this study, we demonstrated a critical role of VEGFR2 in the formation of neovascularization such as VM and new vessels in growing tumors initiated by GSLCs both in xenograft models and in primary GBMs. We observed the presence of tubular vessels formed by tumor cells in human GBMs in which the interface of tumor cell-lined vessels and EC-lined vessels was detected. Tumor cells located in the walls of blood vessels became a part of the vessel surface with the remaining part covered by ECs, a typical structure of “mosaic vessels” [37]. Such “mosaic vessels” have been interpreted as the result of incorporation of the vessel wall by tumor cells [37]. Our study suggests that such tumor cells are likely originated from CSCs with the capability of multi-lineage differentiation including transdifferentiation into EC-like cells that are directly involved in the different phases of neovascularization in growing tumors. In addition to being closely located near microvessels to promote angiogenesis by producing VEGF [19], [21], our present study shows that GSLCs express EC-associated genes coding for EphA2 (ephrin receptor), VE-cadherin, laminin5γ2, and VEGFR-2. These molecules are not only required for the formation and maintenance of blood vessels [38], [39], but are also critical for nervous system development [40], in which neural and vascular guidance pathways share common signaling mechanisms [41]. Therefore, GSLCs with neurodevelopmental potential may utilize common neural and vascular patterning properties to develop blood vessel networks in tumors.

Our study also provided the evidence for the capacity of CD133^+^ GSLCs to form VM and EC-lined vessel by preferentially expressing VEGFR-2, which is required for the self-renewal of GSLCs and tumor initiation. VEGFR-2 (also known as KDR or Flk-1) is a high-affinity receptor for VEGF and is the most important molecule for angiogenesis and vasculogenesis by mediating almost all cellular response to VEGF [42]. Moreover, VEGFR-2 is not only expressed in blood and lymph vessel ECs to promote their recruitment and proliferation [43], but is also expressed in normal stem cells to mediate vasculogenesis [44], [45]. In addition, VEGFR-2 is expressed in tumor cells from patients with colorectal cancer (CRC) [46] and its activation by VEGF in an autocrine and paracrine manner promotes tumor growth [35].

We found that VEGFR-2 is expressed by human GBM cells with a much higher level in CD133^+^ GSLCs, suggesting a link between VEGFR-2 and the biological behavior of GSLCs. Indeed, it has been demonstrated that VEGFR-2 is a critical GSLC-dependent VM biomarker for predisposition of human malignant gliomas to increased patient mortality [9]. Furthermore, functional VEGFR-2 is required by GSLCs not only for VM formation and transdifferentiation into EC, but also for more rapid tumor growth. We speculated that the VM may provide the sufficient nutrition to promote the tumor growth in early date before the appearance of EC-lined vessels. This provides a potential explanation for previously established correlation between the presence of VM in GBM and poor patient prognosis [9].

The demonstration that VEGFR-2 is required for the growth of tumors initiated by GSLCs defines a novel function of VEGFR-2 to control a variety of GSLC properties including self-renewal, multi-lineage differentiation, tumor initiation and angiogenesis [19]–[21], [47], [48]. Such properties are also used by GSLCs for evasion of host anti-tumor immunity [49], [50]. Although it is believed that tumor neovasculature is derived from pre-existing blood vessels or bone-marrow-derived circulating EC progenitors [51], [52], the frequency of BM-derived ECs is very low in tumor neovasculature [53], suggesting that tumor vasculature may be derived either from adjacent existing vessels or from a subpopulation of tumor cells with EC trans-differentiation potential [10], [54]. Our study provides direct evidence that VEGFR-2^+^/CD133^+^ GSLCs serve as progenitors for VM formation and EC-lined vessels. This conclusion is supported by the facts that VEGFR-2^+^/CD133^+^ GSLCs are capable of differentiating into EC and EC-like cells; that some CD133^+^ GSLCs constitutively express both VEGFR-2 and VEGF, which promote their differentiation into EC-like cells in an autocrine or paracrine manner; that blood vessels and VM in xenograft tumors initiated by VEGFR-2^+^/CD133^+^ GSLCs are mainly derived from transplanted human tumor cells; and that the vasculature in xenograft tumors formed by GSLCs containing VEGFR-2 shRNA was formed mostly by mouse-derived ECs.

In the final stage of the preparation of our manuscript, a same research group reported the capacity of glioblastoma cells, and GSLC to form VM in tumors [26], [27], which was independently confirmed by our study. The observation of GSLCs as progenitors for VM formation may explain the reason for generally ineffective anti-angiogenic cancer therapy in the clinic [55]. Recently, the proof-of-the principle has been established for GSLCs as the potential therapeutic targets. The discovery of the novel role of VEGFR-2 in GSLCs for tumorigenicity and vasculogenesis is important for design of additional GSLC-targeting therapies. In this respect, shRNA knockdown of VEGFR-2 reduced the self-renewal, tumor initiation and vascularization, in particular VM formation by GSLCs, has shown promising utilization of such approach for clinical therapies. In addition, the signaling molecules coupled to VEGFR-2 in GSLCs are also relevant to therapeutic design. For instance, VE-cadherin is an EC-specific adherence junction component involved in the formation of complex with VEGFR-2. Elimination of VE-cadherin disrupts VEGF-induced signal transduction necessary for EC survival [56]. In our study, VEGFR-2 knockdown in GSLCs diminished the expression of VE-cadherin in association with reduced formation of VM. Further studies focusing on VEGFR-2-dependent pro-tumorigenic and vasculogenic interactions of GSLCs with VEGF-producing malignant or nonmalignant host cells in the tumor microenvironment will provide insight into the mechanistic basis of tumor development and therapy design.

## Supporting Information

Figure S1 **Enrichment of GSLCs from a human GBM cell line U87. (A)** U87 GBM cell-derived spheres in culture with or without FCS. * Indicates significantly reduced formation of spheres by U87 cells in the presence of FCS in culture (*p<0.01*). **(B)** U87 cells were cultured in stem cell medium containing EGF, b-FGF and supplementary B27 to enrich GSLCs that form floating spheres. The sphere cells express CD133, Nestin, Oct4, Notch1 and Nanog. Scale bar = 20 µm. **(C)** When cultured in the medium with FCS, the floating spheres show the capacity of multi-lineage differentiation by growing in monolayer and expressing microtubule associated protein-2 (MAP2), β-tubulin III and GFAP. Nuclei were counterstained with DAPI. Scale bar = 20 µm.
(TIF)

Figure S2 **Tumor formation by U87 GBM cells and GSLCs in nude mice. (A)** GSLCs and U87 cells were subcutaneously injected into nude mice, which were sacrificed after 4 weeks to obtain tumors. **(B)** Different concentrations of GSLCs were implanted into the flanks of nude mice, which were scarified after 4 weeks to obtain tumors. **(C)** Co-expression of CD133 and VEGFR2 in tumors formed by GSLCs and U87 cells. * Indicates significantly increased number of positive cells in GSLC-formed tumors compared to U87-cell formed tumors (*p<0.05*).
(TIF)

Figure S3 **The effect of VEGFR-2 shRNA on the tumorigenicity of U87 GBM cells.** **(A)** shRNA knockdown of VEGFR-2 in U87 GBM cells. RT-PCR of VEGFR-2 mRNA (*top*); Western blot of VEGFR-2 protein (*bottom*). VEGFR-2 (230 and 200 KDa) and β-actin (an internal control) are indicated. **(B)** Chemotaxis of U87 GBM cells with VEGFR-2 knockdown in response to 10 ng/ml VEGF (*left*) or to different doses of VEGF (*right*). * Indicates significantly reduced chemotaxis of VEGFR-2 knockdown U87 cells in response to VEGF as compared with mock cell chemotaxis (*p<0.05*). **(C)** Proliferation of U87 GBM cells with VEGFR-2 shRNA. * Indicates significantly reduced proliferation of U87 cells with VEGFR-2 shRNA. **(D)** Xenograft tumor growth in nude mice. U87 GBM cells with VEGFR-2 shRNA (2×10^6^) or U87 GBM cells with mock shRNA (2×10^6^) were subcutaneously injected into nude mice (5 mice/group). Tumor growth was monitored up to 52 days. *Indicates significantly reduced growth of tumors formed by U87 cells containing VEGFR-2 shRNA (*p<0.05*). **(E)** Survival rate of mice with xenograft tumors derived from U87 GBM cells with VEGFR-2 shRNA or mock shRNA (5 mice/group). * Indicates significantly prolonged survival of mice bearing VEGFR-2 knockdown U87 cells (*p<0.05*).
(TIF)

Table S1 **Primers used for RT-PCR.** RT-PCR was performed with 0.3 µg total RNA for each sample. The conditions were 30 min at 50°C for reverse transcription, 5 min at 95°C for denaturation and then followed by amplification and extension.
(DOC)

Table S2 **Primers used for qRT-PCR.** All reactions were completed in a 20 µl reaction volume in triplicate and the amplification consisted of 15 min at 42°C for reverse transcription, 5 min at 95°C for denaturization followed by 30 s at 95°C, 1 min at 57°C and 1 min at 72°C for 35 cycles and 10 min at 72°C for extension.
(DOC)
