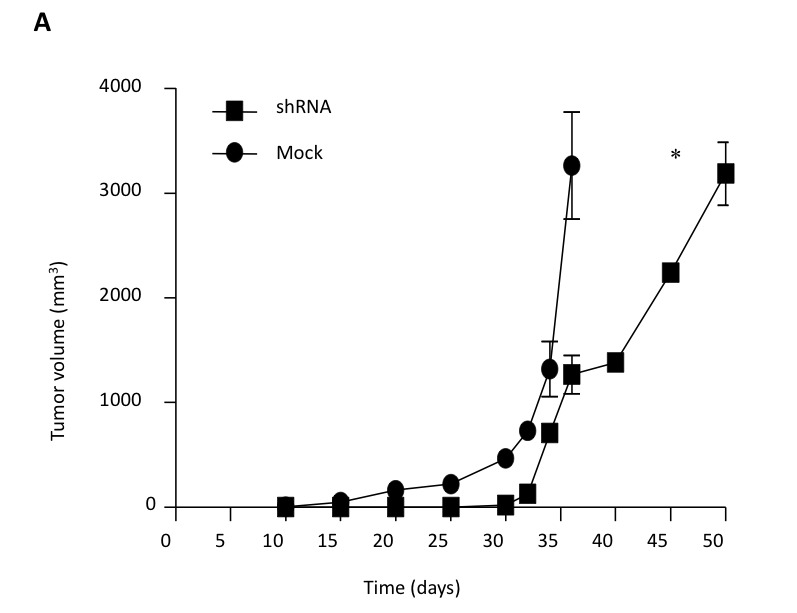# Correction: Vascular Endothelial Growth Factor Receptor 2 (VEGFR-2) Plays a Key Role in Vasculogenic Mimicry Formation, Neovascularization and Tumor Initiation by Glioma Stem-like Cells

**DOI:** 10.1371/annotation/aed5b555-b826-4591-8aa6-284ad888627d

**Published:** 2013-12-30

**Authors:** Xiaohong Yao, Yifang Ping, Ying Liu, Kequiang Chen, Teizo Yoshimura, Mingyong Liu, Wanghua Gong, Chong Chen, Qin Niu, Deyu Guo, Xia Zhang, Ji Ming Wang, Xiuwu Bian

In Figure 6A, the labels "shRNA" and "Mock" have been switched. Please see the corrected Figure 6A here: 

**Figure:**