# Supplementary figures and images for: Vascular Endothelial Growth Factor Receptor 2 (VEGFR-2) Plays a Key Role in Vasculogenic Mimicry Formation, Neovascularization and Tumor Initiation by Glioma Stem-like Cells
Source: PLoS One. 2013 Mar 11;8(3):e57188. doi: 10.1371/journal.pone.0057188 (PMC3594239; doi:10.1371/journal.pone.0057188)

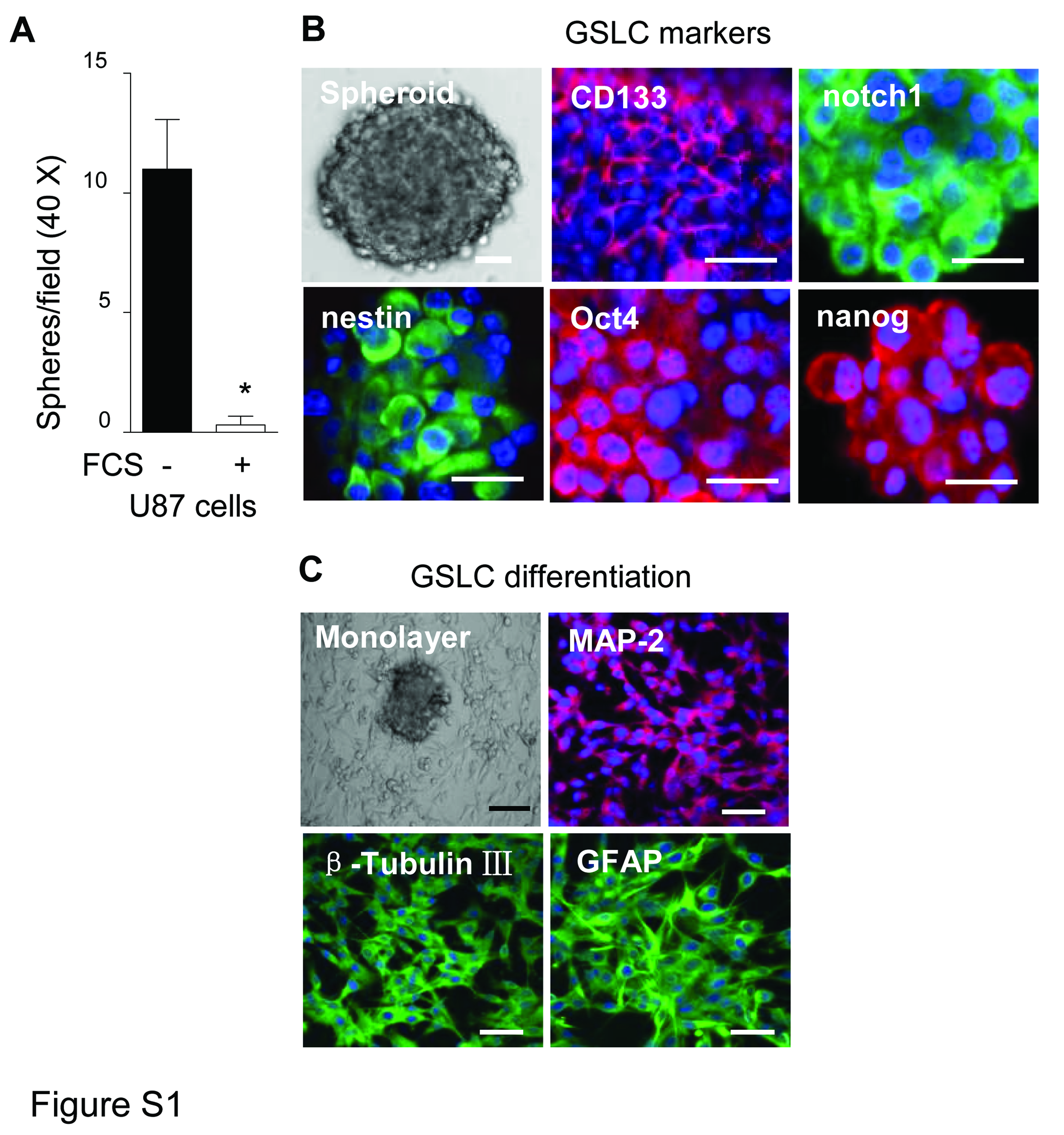

Supplement: Figure S1 — Enrichment of GSLCs from a human GBM cell line U87. (A) U87 GBM cell-derived spheres in culture with or without FCS. * Indicates significantly reduced formation of spheres by U87 cells in the presence of FCS in culture (p<0.01). (B) U87 cells were cultured in stem cell medium containing EGF, b-FGF and supplementary B27 to enrich GSLCs that form floating spheres. The sphere cells express CD133, Nestin, Oct4, Notch1 and Nanog. Scale bar = 20 µm. (C) When cultured in the medium with FCS, the floating spheres show the capacity of multi-lineage differentiation by growing in monolayer and expressing microtubule associated protein-2 (MAP2), β-tubulin III and GFAP. Nuclei were counterstained with DAPI. Scale bar = 20 µm. (TIF) [file pone.0057188.s001.tif]

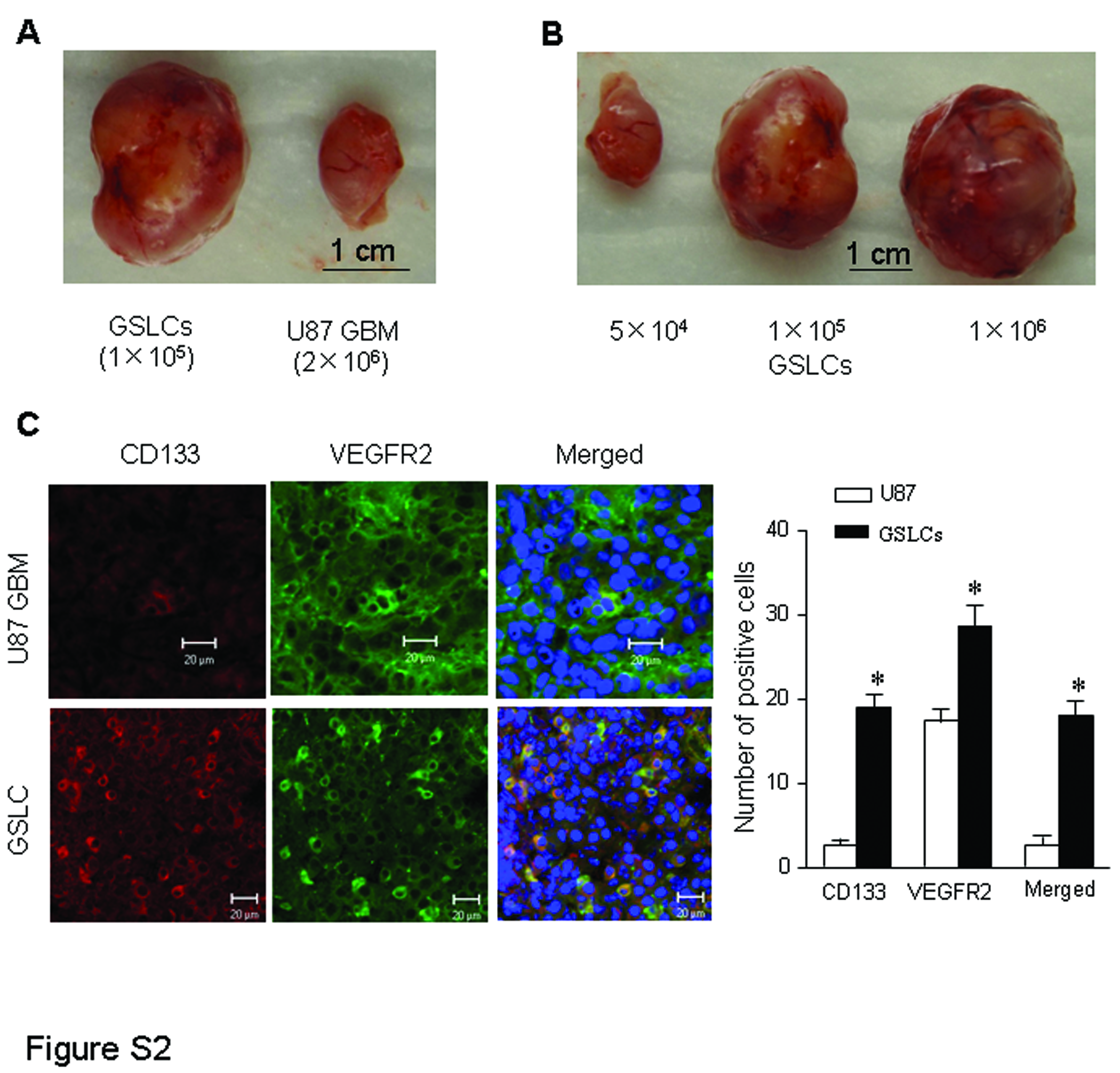

Supplement: Figure S2 — Tumor formation by U87 GBM cells and GSLCs in nude mice. (A) GSLCs and U87 cells were subcutaneously injected into nude mice, which were sacrificed after 4 weeks to obtain tumors. (B) Different concentrations of GSLCs were implanted into the flanks of nude mice, which were scarified after 4 weeks to obtain tumors. (C) Co-expression of CD133 and VEGFR2 in tumors formed by GSLCs and U87 cells. * Indicates significantly increased number of positive cells in GSLC-formed tumors compared to U87-cell formed tumors (p<0.05). (TIF) [file pone.0057188.s002.tif]

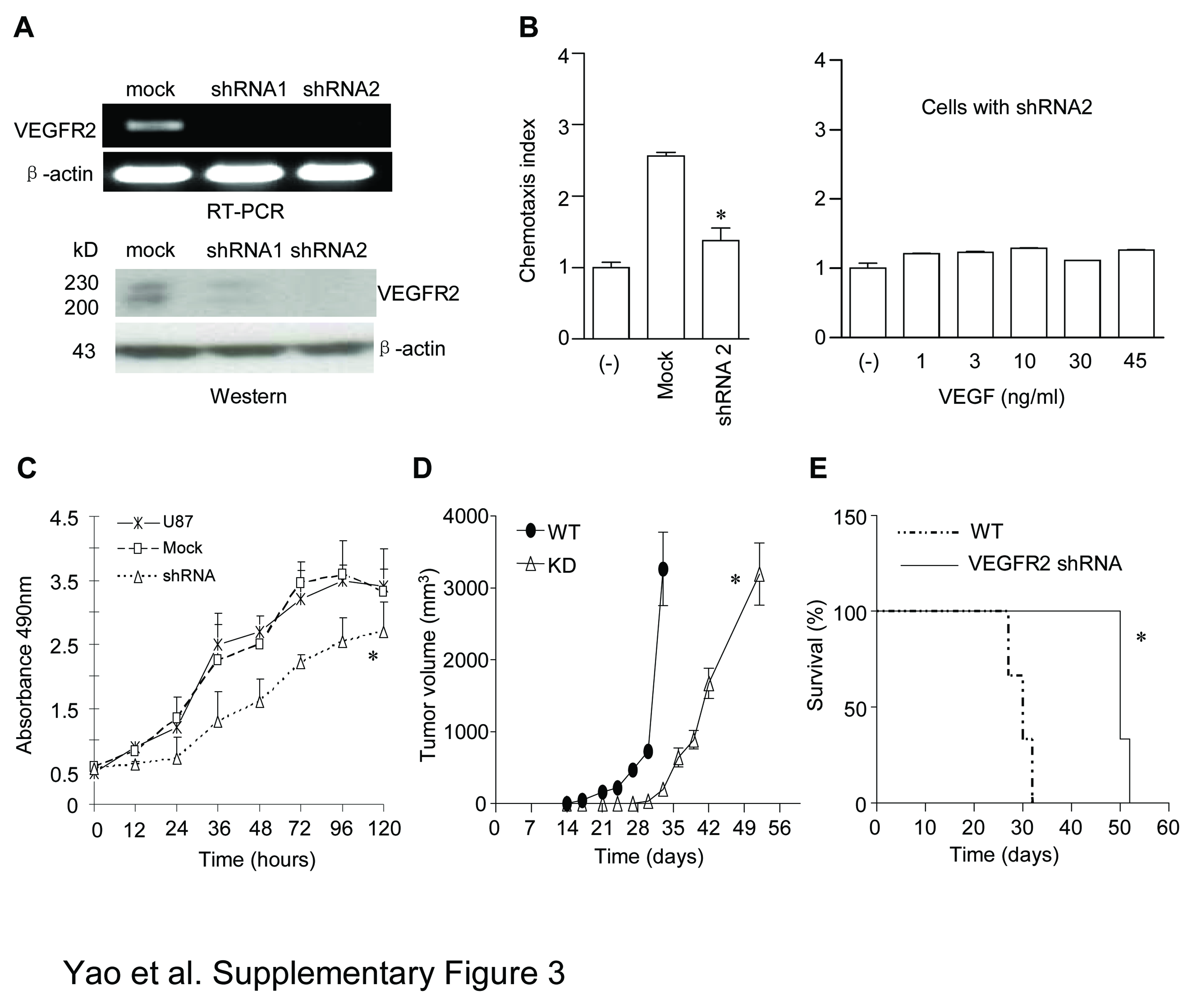

Supplement: Figure S3 — The effect of VEGFR-2 shRNA on the tumorigenicity of U87 GBM cells. (A) shRNA knockdown of VEGFR-2 in U87 GBM cells. RT-PCR of VEGFR-2 mRNA (top); Western blot of VEGFR-2 protein (bottom). VEGFR-2 (230 and 200 KDa) and β-actin (an internal control) are indicated. (B) Chemotaxis of U87 GBM cells with VEGFR-2 knockdown in response to 10 ng/ml VEGF (left) or to different doses of VEGF (right). * Indicates significantly reduced chemotaxis of VEGFR-2 knockdown U87 cells in response to VEGF as compared with mock cell chemotaxis (p<0.05). (C) Proliferation of U87 GBM cells with VEGFR-2 shRNA. * Indicates significantly reduced proliferation of U87 cells with VEGFR-2 shRNA. (D) Xenograft tumor growth in nude mice. U87 GBM cells with VEGFR-2 shRNA (2×10^6^) or U87 GBM cells with mock shRNA (2×10^6^) were subcutaneously injected into nude mice (5 mice/group). Tumor growth was monitored up to 52 days. *Indicates significantly reduced growth of tumors formed by U87 cells containing VEGFR-2 shRNA (p<0.05). (E) Survival rate of mice with xenograft tumors derived from U87 GBM cells with VEGFR-2 shRNA or mock shRNA (5 mice/group). * Indicates significantly prolonged survival of mice bearing VEGFR-2 knockdown U87 cells (p<0.05). (TIF) [file pone.0057188.s003.tif]
