# Supplementary material for: Vascular Endothelial Growth Factor Receptor 2 (VEGFR-2) Plays a Key Role in Vasculogenic Mimicry Formation, Neovascularization and Tumor Initiation by Glioma Stem-like Cells
Source: PLoS One. 2013 Mar 11;8(3):e57188. doi: 10.1371/journal.pone.0057188 (PMC3594239; doi:10.1371/journal.pone.0057188)
Supplement: Table S1 — Primers used for RT-PCR. RT-PCR was performed with 0.3 µg total RNA for each sample. The conditions were 30 min at 50°C for reverse transcription, 5 min at 95°C for denaturation and then followed by amplification and extension. (DOC) [file pone.0057188.s004.doc]

**Table S1. Primers used for RT-PCR**

| Gene sequence of primers (5’-3’) | Amplification and extension | Product length (base pairs) |
| --- | --- | --- |
| VEGFR1  For: TGGGACAGTAGAAAGGGCTT  Rev: GGTCCACTCCTTACACGACAA | 95°C 30 s, 57°C 1 min, 72°C 1 min for 35 cycles and then 10 min at 72°C | 395 |
| VEGFR2  For: CCGTCAAGGGAAAGACTACG  Rev: CTTTACCCCAGGATATGGAG | 95°C 30 s, 58°C 30 s, 72°C 1 min for 35 cycles and then 10 min at 72°C | 496 |
| VE-cadherin  For: ACCGGATGACCAAGTACAGC  Rev: ACACACTTTGGGCTGGTAGG | 95°C 1 min, 55°C 45 s, 72°C 1 min for 35 cycles and then 10 min at 72°C | 592 |
| CD31  For: TCCGGATCTATGACTCAGGG  Rev: ACAGTTGACCCTCACGATCC | 94°C 30 s, 60°C 1 min, 72°C 1 min 30 s for 35 cycles and then 7 min at 72°C | 151 |
| Epha2  For: ATGGAGCTCCAGGCAGCCCGC  Rev: GCCATACGGGTGTGTGAGCCAGC | 94°C 30 s, 60°C 1 min, 72°C 1 min 30 s for 35 cycles and then 7 min at 72°C | 147 |
| Laminin 5γ2  For: AGCAGAAAGCCACGTTGAGT  Rev: CAGGGACTTGGTTTTCTCCA | 94°C 30 s, 60°C 1 min, 72°C 1 min 30 s for 35 cycles and then 7 min at 72°C | 187 |

RT-PCR was performed with 0.3 μg total RNA for each sample. The conditions were 30 min at 50°C for reverse transcription, 5 min at 95°C for denaturation and then followed by amplification and extension.
