# Supplementary material for: Vascular Endothelial Growth Factor Receptor 2 (VEGFR-2) Plays a Key Role in Vasculogenic Mimicry Formation, Neovascularization and Tumor Initiation by Glioma Stem-like Cells
Source: PLoS One. 2013 Mar 11;8(3):e57188. doi: 10.1371/journal.pone.0057188 (PMC3594239; doi:10.1371/journal.pone.0057188)
Supplement: Table S2 — Primers used for qRT-PCR. All reactions were completed in a 20 µl reaction volume in triplicate and the amplification consisted of 15 min at 42°C for reverse transcription, 5 min at 95°C for denaturization followed by 30 s at 95°C, 1 min at 57°C and 1 min at 72°C for 35 cycles and 10 min at 72°C for extension. (DOC) [file pone.0057188.s005.doc]

**Table S2. Primers used for qRT-PCR**

|  | Forward primer | Reverse primer | Product length (base pairs) |
| --- | --- | --- | --- |
| VEGFR2 | GCAGGGGACAGAGGGACTTG | GAGGCCATCGCTGCACTCA | 91 |
| VE-cadherin | TCGTCATGGACCGAGGTT | TCTACAATCCCTTGCAGTGTGA | 131 |
| CD31 | TCCGGATCTATGACTCAGGG | ACAGTTGACCCTCACGATCC | 151 |
| Epha2 | ATGGAGCTCCAGGCAGCCCGC | GCCATACGGGTGTGTGAGCCAGC | 147 |
| Laminin 5γ2 | AGCAGAAAGCCACGTTGAGT | CAGGGACTTGGTTTTCTCCA | 187 |
| β-actin | CGGGA AATCTGCGTGACATT | GGAGTTGAAGGTAGTTTCGTGG | 210 |

All reactions were completed in a 20 μl reaction volume in triplicate and the amplification consisted of 15 min at 42°C for reverse transcription, 5 min at 95°C for denaturization followed by 30 s at 95°C, 1 min at 57°C and 1 min at 72°C for 35 cycles and 10 min at 72°C for extension.
